# Microplastic in angling baits as a cryptic source of contamination in European freshwaters

**DOI:** 10.1038/s41598-021-90468-0

**Published:** 2021-05-27

**Authors:** Aline Reis de Carvalho, Alexis Imbert, Ben Parker, Axelle Euphrasie, Stéphanie Boulêtreau, J. Robert Britton, Julien Cucherousset

**Affiliations:** 1 CNRS, Université Toulouse III - Paul Sabatier, IRD, UMR 5174 Laboratoire Évolution et Diversité Biologique (EDB), 118 route de Narbonne, 31062 Toulouse, France; 2 CNRS, Université Toulouse III - Paul Sabatier, UMR 5623 Laboratoire des Interactions Moléculaires et Réactivité Chimique et Photochimique (IMRCP), 118 route de Narbonne, 31062 Toulouse, France; 3 Department of Life and Environmental Sciences, Faculty of Science and Technology, Bournemouth University, Fern Barrow, Poole, Dorset, BH12 5BB UK; 4 CNRS, Université Toulouse III - Paul Sabatier, UMR 5245 Laboratoire Écologie Fonctionnelle et Environnement, 118 route de Narbonne, 31062 Toulouse, France

**Keywords:** Environmental impact, Environmental monitoring

## Abstract

High environmental microplastic pollution, and its largely unquantified impacts on organisms, are driving studies to assess their potential entry pathways into freshwaters. Recreational angling, where many anglers release manufactured baits into freshwater ecosystems, is a widespread activity with important socio-economic implications in Europe. It also represents a potential microplastic pathway into freshwaters that has yet to be quantified. Correspondingly, we analysed three different categories of industrially-produced baits (‘groundbait’, ‘boilies’ and ‘pellets’) for their microplastic contamination (particles 700 µm to 5 mm). From 160 samples, 28 microplastics were identified in groundbait and boilies, with a mean concentration of 17.4 (± 48.1 SD) MP kg^−1^ and 6.78 (± 29.8 SD) mg kg^−1^, yet no microplastics within this size range were recorded in the pellets. Microplastic concentrations significantly differed between bait categories and companies, but microplastic characteristics did not vary. There was no correlation between microplastic contamination and the number of bait ingredients, but it was positively correlated with C:N ratio, indicating a higher contamination in baits with higher proportion of plant-based ingredients. We thus reveal that bait microplastics introduced accidentally during manufacturing and/or those originating from contaminated raw ingredients might be transferred into freshwaters. However, further studies are needed to quantify the relative importance of this cryptic source of contamination and how it influences microplastic levels in wild fish.

## Introduction

Microplastic pollution (plastic particles < 5 mm in size) represents a growing and ubiquitous threat to ecosystems^1,2^. In freshwater, microplastics primarily originate from the fragmentation of larger plastic items^3^, and their prevalence in lakes^4,5^ and rivers^6,7^ can be high. Microplastics are consumed by aquatic organisms across trophic levels and taxa^8–10^, representing toxicological threats to individuals and subsequently affecting community composition and the functioning of freshwater ecosystems^11,12^. Microplastic characteristics, such as colour and shape, can modulate their consumption by aquatic organisms^13–15^, with their consumption being either direct (occurring both intentionally or accidently^15^) or indirect through the consumption of food resources contaminated with microplastics^16,17^. Identifying microplastic sources and their pathways into freshwater ecosystems is therefore important for reducing their potential impacts^18^.

Angling is a widespread recreational activity practiced by more than 10% of the global population^19^ and by up to 20% of populations in some European countries^20^. While angling is multifaceted in the way anglers capture a fish, most techniques release angling baits into the water to attract fish into a restricted spatial area in order to maximise the chance of fish capture^21^. Baits are introduced into freshwater ecosystems by anglers, either by hands and/or using devices such as a catapult. Anglers use, on average, 7.3 kg of baits per year^22^, with some specialised anglers using at least 200 kg of angling bait per year^23^. Evidence suggests that angling baits can represent an important food resource to wild fish, contributing to over half of the diet of fish in some ecosystems, and nearly 80% for some individuals^24^. This dietary contribution by bait tends to increase with fish size^25^, with larger specimens often being targeted by anglers more than smaller fish and they can be more vulnerable to capture^26^. Angling baits represent important trophic subsidies to freshwater ecosystems that can additionally contribute to eutrophication through the addition of phosphorous^27,28^.

Angling baits are usually purchased by anglers from commercial sources and can be categorised as ‘groundbaits’ (composed of relatively fine particles, often used for attracting smaller fish and mixed with water to obtain a compact ball), ‘boilies’ (circular, hardened baits of usually up to 24 mm diameter that are designed to select for larger fish) and ‘pellets’ (usually pelletized fish meal products of 3 to 24 mm diameter). These angling baits differ in their composition but generally contain various flours (plant- and/or animal-based) mixed with additional ingredients. Because commercially-available angling baits are primarily produced industrially, there is potential that they also contain substantial quantities of microplastics, either present in the raw materials or introduced during manufacture. Microplastics have already been reported in other industrially-produced and packaged wines^29^, pet foods^30^ and canned fish for human consumption^31,32^. Therefore, angling baits could represent an unknown pathway of microplastic contamination within freshwater ecosystems that requires quantification, especially given their ubiquitous use in angling in many European countries^23,28^.

This study aimed to investigate the presence of microplastics within angling baits as a potential source of microplastic to freshwater pollution. The objectives were to firstly quantify the number, mass and characteristics (size, colour and polymeric composition) of microplastics within commercially-available, industrially-produced, angling baits (several products of three main bait categories: groundbaits, boilies and pellets), and to determine if contamination levels varied between bait categories and companies. This study also explored whether differences in microplastic number or characteristics could be related to the number of ingredients, and the origin of ingredients. The latter was assessed using the carbon to nitrogen (C:N) ratio to determine the relative amount of animal- versus plant-based ingredients (smaller C:N ratio with high proportion of animal-based ingredients)^33,34^. Specifically, we tested the hypothesis that (1) the number of ingredients in angling baits was positively related to microplastic concentration, as it likely represents an increased diversity of the potential sources of contamination; and (2) the ingredients of the angling baits are a major determinant of their microplastic concentration, with animal-based baits containing more microplastic than plant-based baits.

## Results

### Microplastic contamination levels

**Figure 1:**
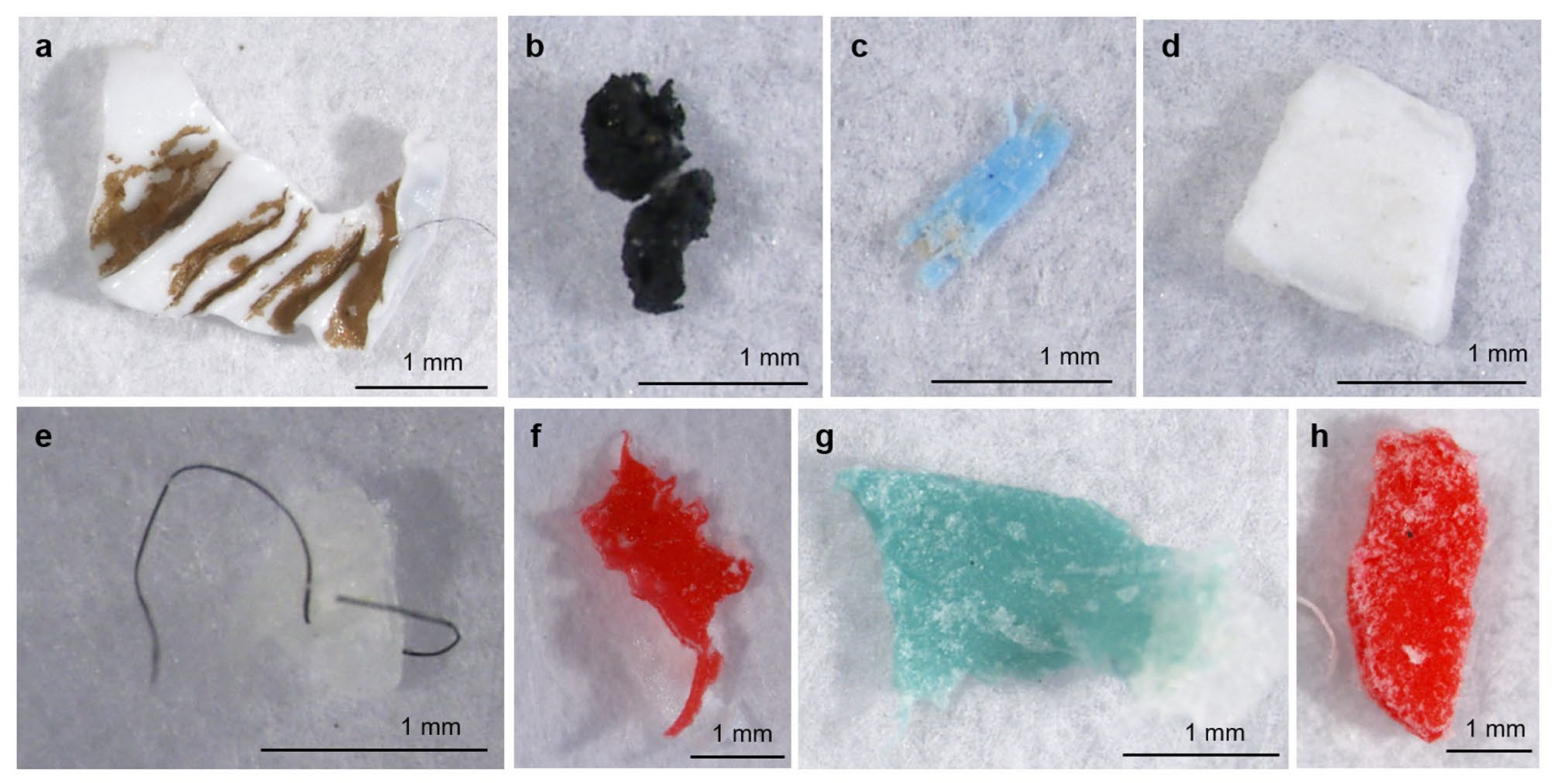
Examples of microplastics particles (colour, polymer composition and shape) found in angling baits (category, G = groundbait and B = boilies, and product, 1–6): (**a**) white polypropylene fragment (B6); (**b**) black additive fragment (G1); (**c**) blue polyethylene fragment (B2); (**d**) white additive fragment (B2); (**e**) black additive fibre (G1); (**f**) red polyethylene fragment (G2); (**g**) blue polyethylene fragment (G6) and (**h**) red polyethylene fragment (G6).

Microplastics (700 µm–5 mm) were investigated within 16 commercially available angling baits products (6 groundbaits, 6 boilies and 4 pellets) that were purchased in France, with each bait replicated 10 times. Across the 160 analysed samples, a total of 86 particles were collected. Infrared spectroscopy analyses revealed that 39 particles were plastic, of which 28 were within the selected size range of microplastics, i.e. 700 µm to 5 mm (Fig. 1 and Supplementary Figure S1). Therefore, 11 plastic particles were excluded from further analyses, and 20 microplastics were collected in groundbaits and 8 in boilies. Correspondingly, microplastic contamination of pellets within the selected size range was considered as null.

**Figure 2:**
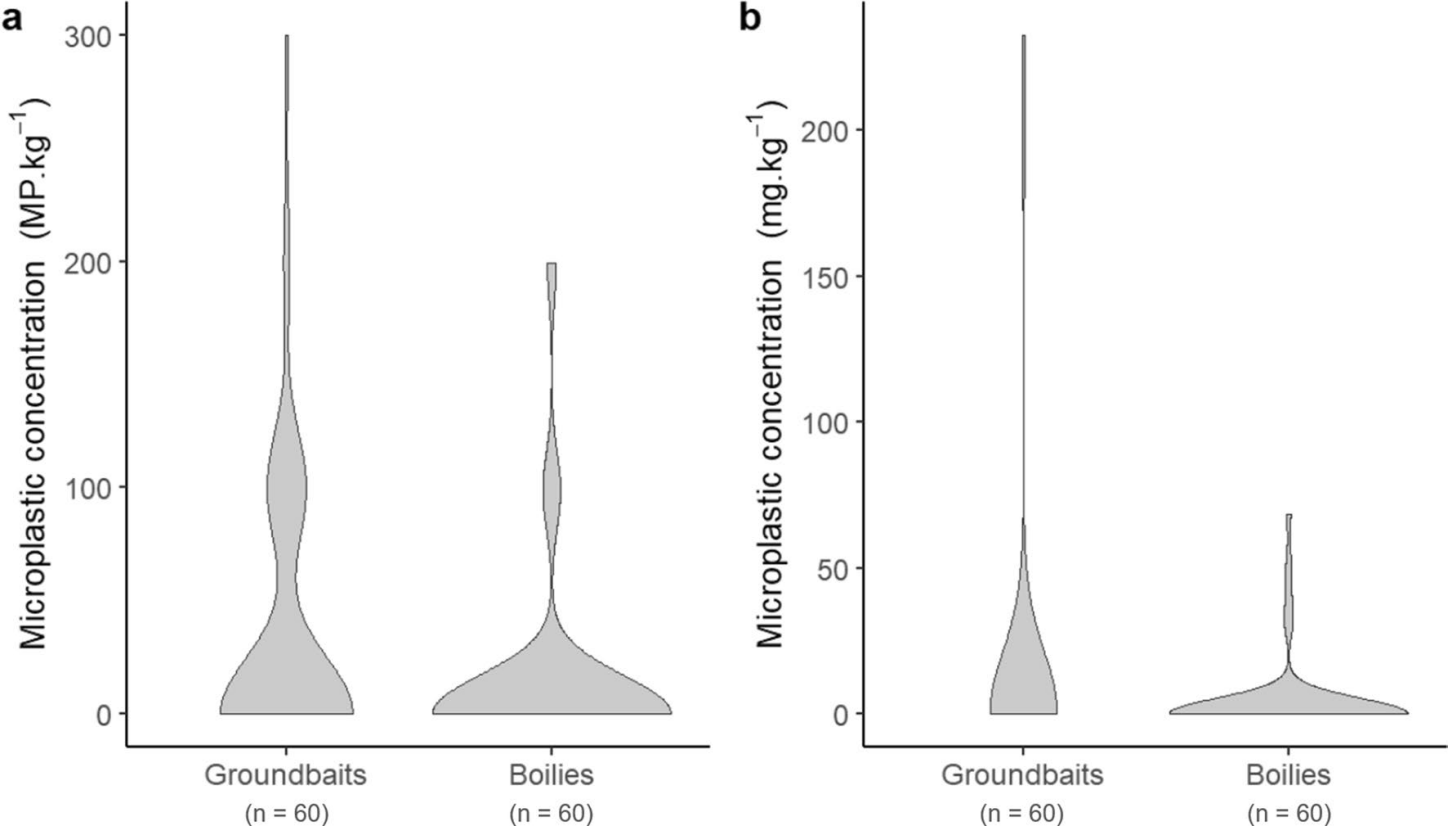
Microplastic concentrations in angling baits in (**a**) number (MP kg^−1^) and (**b**) mass (mg kg^−1^).

The mean occurrence of microplastics across all angling bait samples was 13.7 ± 17.01%, with occurrence varying between bait categories (groundbait: 26.7 ± 19.7%; boilies: 10.0 ± 10.9%). There was a significant difference in the occurrence of microplastics between bait categories (Fisher Test, *p* = 0.032) and companies (Fisher Test, *p* < 0.001). The microplastic concentration level ranged between 0 and 300 MP kg^−1^ (Supplementary Table S1), where the mean concentration over all samples was 17.4 ± 48.1 MP kg^−1^. The difference in MP levels between groundbait and boilies were not significant (groundbait: range 0—300 MP kg^−1^; mean 33.3 ± 62.8 MP kg^−1^; boilies: range: 0 – 199 MP kg^−1^, mean 13.2 ± 42.8 MP kg^−1^, glmm: Χ^2^ = 15,468.2, *p* < 0.001, post-hoc pairwise comparison, *p* = 0.082, Fig. 2a). A significant difference in MP levels was detected between companies (glmm: Χ^2^ = 2863.1, *p* < 0.001).

The range in MP concentration by mass across all samples was 0 to 232 mg kg^−1^ (mean 6.78 ± 29.8 mg kg^−1^). There were a significantly higher concentration in groundbaits than in boilies (groundbait: range 0 to 232 mg kg^−1^, mean 14.2 ± 46.1 mg kg^−1^; boilies: range 0 to 68.3 mg kg^−1^, mean 3.91 ± 13.3 mg kg^−1^; glmm: Χ^2^ = 29,758.1, *p* < 0.001, post-hoc pairwise comparison, *p* < 0.001 Fig. 2b). Significant differences were also found between companies (glmm, Χ^2^ = 4027.6, *p* < 0.001).

### Microplastic characteristics

**Figure 3:**
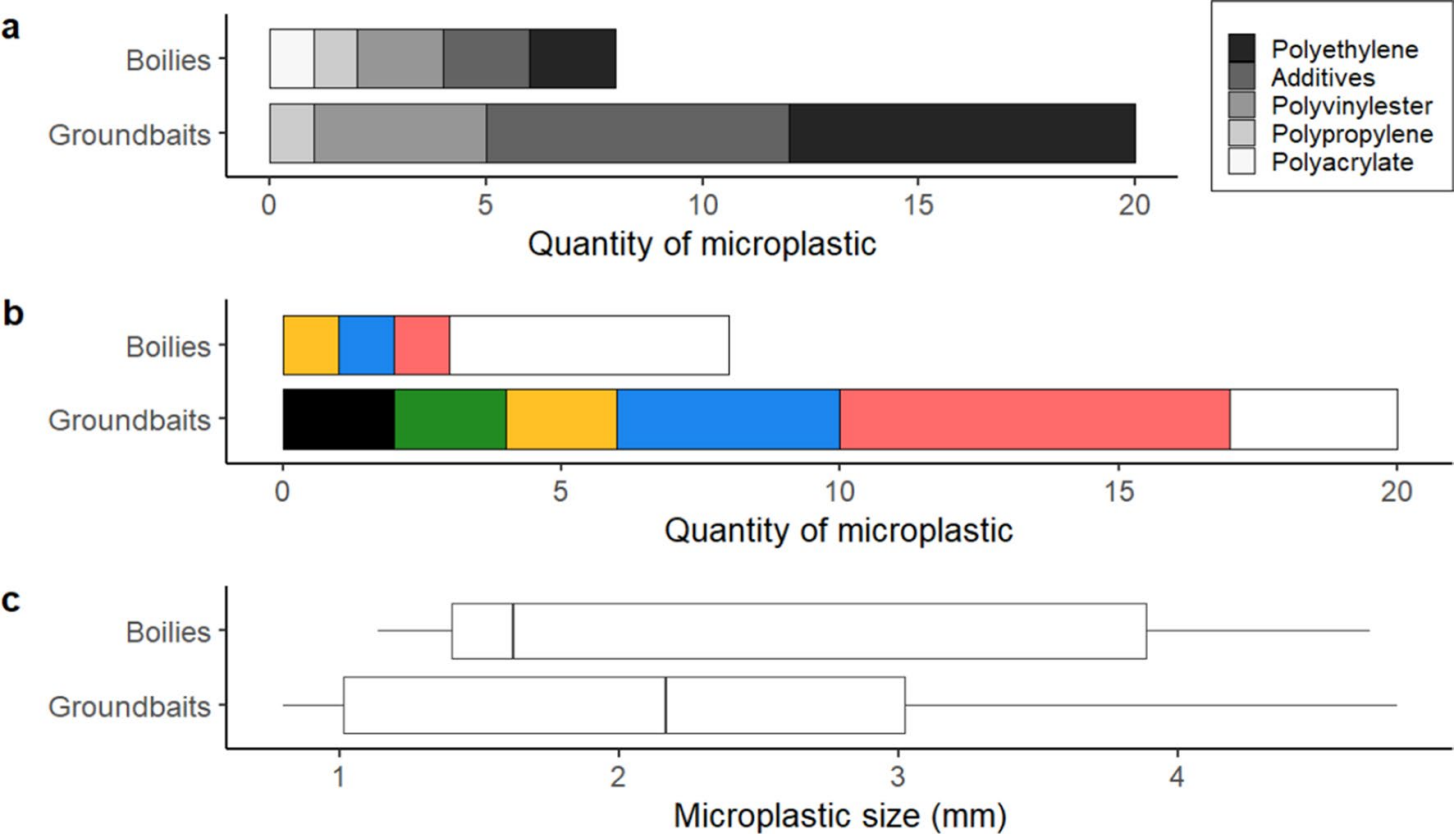
Characteristics of microplastics found in boilies (n  = 8) and groundbaits (n = 20): (**a**) polymer composition, (**b**) colour (as displayed) and (**c**) size (mm).

The microplastics detected in groundbaits and boilies were almost exclusively comprised of fragments, with only one fibre detected (Fig. 1e). Polyethylene was the main polymer found across these two categories (35.7%), followed by artificial additives (32.1%) (mainly alkyd resins), polyvinylester (21.5%), polypropylene (7.1%) and polyacrylate (3.6%) (Fig. 3a). Red and white were the main colours detected (both 28.6%), but with blue (17.9%), yellow (10.7%), black and green (7.1% each) also present (Fig. 3b). The mean MP size was 2.25 ± 1.26 mm (Fig. 3c). Overall, there was no significant difference between groundbaits and boilies in microplastic composition and colour (Fisher tests, *p* > 0.05), and microplastic size (Wilcoxon test, *p* > 0.05).

The bait packaging was composed of two polymers, polyethylene terephthalate and polyethylene (Supplementary Table S2). Polyethylene terephthalate was never detected as a contaminant of angling baits and only 37.5% of packages were polyethylene. Other microplastics present in the baits were also not associated with their packaging (e.g. polyvinylester (G2 and B2) and artificial additives (B2)). Thus, microplastics found in these baits were apparently not primarily derived from their packaging.

### Relationships of microplastic levels with bait ingredients and C:N ratios

**Figure 4:**
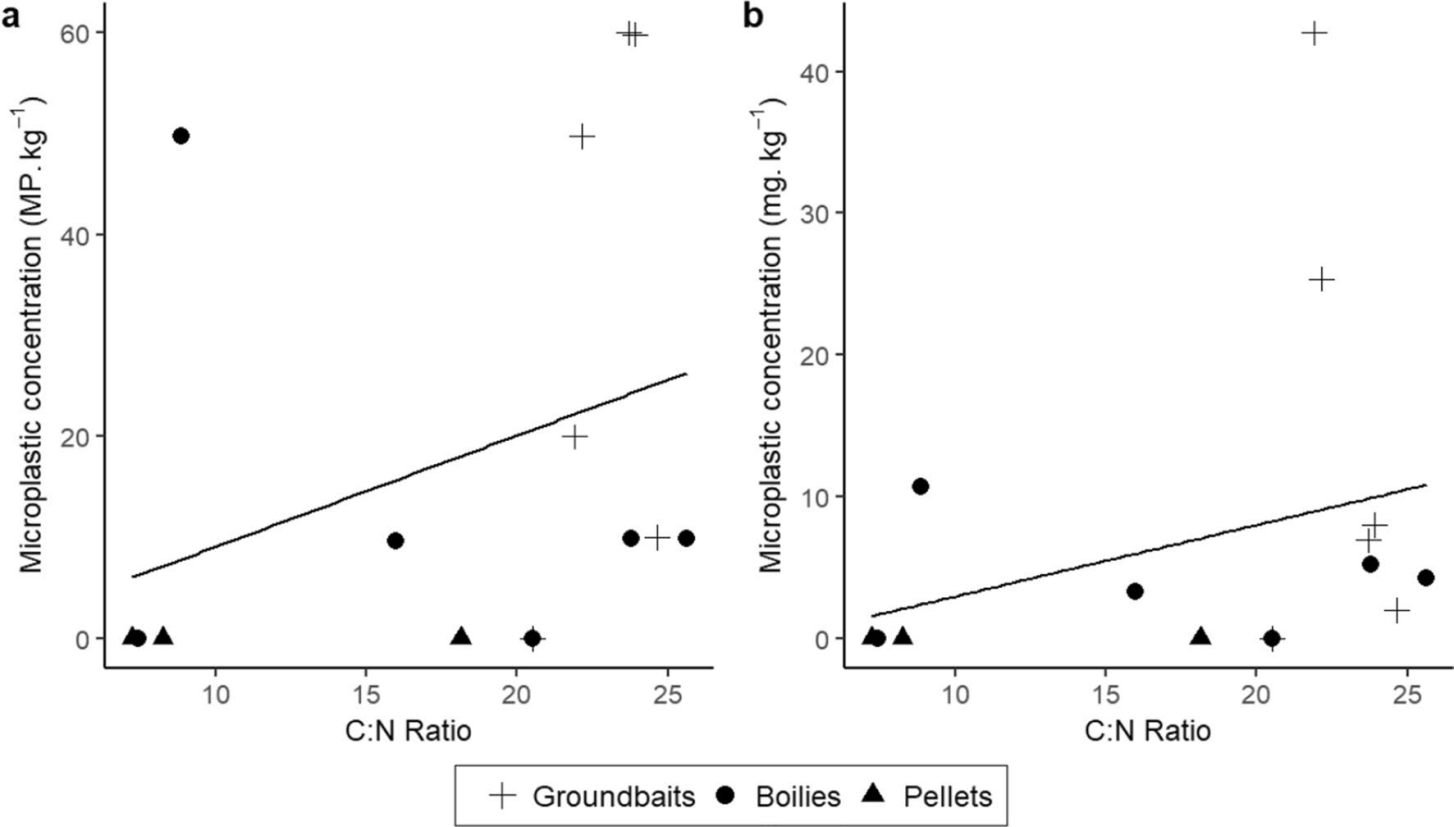
Relationship between average microplastic concentration (across all replicates) in (**a**) number (MP kg^−1^) and (**b**) mass (mg kg^−1^), and C:N ratio in angling baits. A lower C:N ratio indicates a higher proportion of animal-based components.

When analysing all angling products together (n = 16), the correlations between the number of ingredients reported on packages and microplastic concentrations were not significant (Spearman correlations, ρ = 0.15, *p* = 0.572 and ρ = 0.24, *p* = 0.375 for number and mass concentration, respectively) (Supplementary Figure S2). A significant and positive correlation was observed between microplastic concentrations and C:N ratio (Spearman correlations, ρ = 0.62, *p* = 0.018 and ρ = 0.55, *p* = 0.028 for number and mass concentration, respectively) (Fig. 4). This suggests that microplastic concentration was higher in angling baits with higher C:N ratios.

## Discussion

This investigation into microplastic contamination in angling baits revealed microplastic particles (700 µm–5 mm) contaminated two out of three studied bait categories; groundbaits and boilies, with significant differences between categories and companies. Microplastics were mainly composed of polyethylene and artificial additives such as alkyd resins and paint additives, and were mainly white, red and blue. There was no correlation between the number of bait ingredients and their microplastic concentration and the C:N ratio of the baits was positively correlated with contamination level.

Given the incidence and number of microplastics per unit of bait mass, they could represent a significant source of microplastics to freshwater fish when fishing pressure is high. Once in the water, fish may consume the microplastics derived from angling baits either directly, i.e. microplastic released from bait, or indirectly through the ingestion of contaminated bait or other biota that have themselves consumed bait. The fact that anglers tend to target larger individuals^26^, whose diets may also depend heavily on angling baits^24,25^, suggests that larger fish might be most exposed to microplastics via this pathway. While several studies have already identified correlations between fish body size and microplastic loads in fish^35–39^, it is currently unknown whether this might be related to the consumption of contaminated baits.

Species of the Cyprinidae family are the primary target for the angling baits investigated and microplastics have previously been detected in common carp (*Cyprinus carpio*) from several rivers^40–43^. Microplastic incidence and counts within cyprinid fishes have generally been high compared to other fish within the same system, ranging from 2.5 to 48 pieces per individual in the gastrointestinal tract^41–43^, with many of these studies implicating the benthic foraging habits of carp as a likely explanation. Fish feeding largely on baits, particularly large individuals, could reasonably achieve the levels of microplastic contamination observed in the wild, although it is acknowledged that identifying the sources of specific microplastics is difficult. While the potential effects of ingestion of contaminated baits have also yet to be determined, microplastic exposure has been shown to adversely affect *C. carpio* biochemistry, immunological activity, growth and oxidative pathways within the laboratory^44–46^.

The diverse size range of microplastic particles detected in studies limits comparisons between the microplastic concentrations in the baits and those that are generally found in the biota^47^. However, comparisons of bait microplastics with those detected in wild fish have shown that both are dominated by particles of varying colours^41,42^. Polymeric compositions of microplastics found in baits were also similar to those in wild fish, with polypropylene and polyethylene common^39,41^, although other studies have also identified polymers such as polytetrafluoroethylene and rayon^42^, which were absent from our angling baits. Polymers composing identified microplastics in the angling baits as polyethylene and polypropylene account for nearly 50% of all plastics demand in Europe (2019)^49^. As such, it is difficult to draw inferences about microplastic contributions via angling baits by comparing polymer data alone.

The absence of microplastics in the selected size range (700 µm–5 mm) in the pelletized angling baits suggests some key differences in their ingredients and/or manufacturing process compared with groundbait and boilies. Polyethylene microplastics and additives such as polyolefin and alkyd resins found in the angling baits are commonly present in machinery paints^50^ that might gradually fragment over time. Various heating, milling and filtering processes during manufacturing may also alter, and fragment microplastic particles, potentially producing smaller particles falling outside the minimal threshold used in the present study^51,52^. This higher level of industrial manufacturing might explain the absence of larger particles in the pelletized angling baits. Considering that particle size is an important factor determining microplastic ingestion and impacts on organisms^47,53^, further investigations focusing on smaller fragment sizes (< 700 µm) are needed. That the microplastics identified in the baits were of a different composition to their packaging further suggests contamination existing within the raw ingredients and/or introduced during their manufacture, although further work is necessary to identify the exact stage(s) and source(s) of contamination.

Commercial fishmeal has previously been shown to contain microplastics^51,52^, with often higher concentrations occurring in those with particular ingredients and/or manufacturing processes. Fishmeal was found to contain mostly fragments 100–1000 µm in size that were composed of polypropylene, polyethylene and polystyrene^51,52^, which are largely comparable to those particles found in our angling baits. Hanachi et al. (2019) additionally found higher microplastic concentrations in salmon and sardine than kilka-derived fishmeal, whereas, contrary to our first hypothesis, we found a positive correlation between bait C:N ratio and microplastic contamination, suggesting lower contamination in animal-based baits. Nevertheless, the similarity in microplastic features to those recovered from industrialized food^29^ suggests at least some procedural contamination from the manufacture process.

The estimates of the extent of cryptic microplastic emissions from angling baits were as high as 0.34 tons per year for a country, when considering 7.3 kg of groundbaits/angler/year and 3.3 million active anglers, as in Germany^28^, but this does largely depend on the activity of anglers in a country and the amounts of baits they apply in their angling. Nevertheless, this estimate is comparable to the annual 0.15 tons of microplastics released through the use of winter de-icing salts applied to roads in some European countries^54^. At a larger scale, the microplastic contribution through angling baits can be considered minor compared to the top sources of European riverine microplastics (tyre wear particles, polymer-based textiles and polymers washed in from road dusts) which may each contribute more than 0.3 kilotons of microplastic a year^55^. Nevertheless, angling bait-derived microplastics may make up a large proportion of local microplastic concentrations in particular locations or at particular times of the year when the baits are heavily used. Also, as expected for natural particles, microplastic concentration might increase with decreasing particle size^56^, and the total contamination by microplastics would probably be substantially higher, as only particles from 700 µm to 5 mm were considered here. Further investigations are needed to fully understand the potential contribution of this cryptic source of microplastic pollution compared to the global microplastic pollution in freshwater ecosystems. This contribution will likely be extremely variable among ecosystems and countries, depending on both the characteristics of the ecosystems (e.g. size, fish community) and of the fisheries (e.g. amount and type of bait used). We posit that in small lakes with intense fishing pressure targeting coarse fishes angling baits might represent an important source of microplastic pollution compared to other sources. Since angling baits are already known to contribute to freshwater eutrophication^27,28^, the additional release of microplastics from contaminated baits may represent another, co-occurring stressor to freshwater systems which requires further investigation. The awareness of hidden sources could contribute to the design of studies investigating consequences and impacts of microplastic to human and animal health.

The ubiquitous presence of microplastic particles in the environment means that mitigating this angling source of contamination might have a negligible impact. Nevertheless, our results are important in highlighting a previously unknown source of microplastic loadings in freshwater fishes and thus have h elped identify a novel source and pathway. Such cryptic sources of microplastic contamination to freshwater ecosystems reveal the ubiquity of plastics within products used in daily hu man activities and, more specifically, on the relevance of angling activity in European countries in increasing the exposure of fish to these plastics. The manufacturing process of industrialized food, either for human or animal consumption, thus represents a potential source of microplastic contamination that has yet to be fully quantified and therefore further studies are encouraged in order to investigate the sources of these cryptic microplastics, and their fates and impacts in the environment.

## Methods

### Bait selection

We purchased some of the most popular, commercially-available, angling baits used in Europe, i.e. groundbaits, boilies and pellets, to target freshwater cyprinid fish. Angling baits were purchased in two angling shops and online from a popular angling website in France. In total, 16 different products were purchased (6 for groundbaits, 6 for boilies and 4 for pellets), produced by 6 different companies, therefore including multiple bait categories from some companies. The products differed from each other by their commercial name or packaging and, in total, 27 commercial bags were purchased (Supplementary Table S1). The elemental composition (carbon and nitrogen) was quantified and the C:N ratio calculated. A relatively high C:N value indicates that the bait has a higher proportion of plant-based ingredients, whereas a relatively low C:N ratio indicates that the bait has a higher proportion of animal-based ingredients^33,34^. About 3 g of each angling bait, in triplicates, was oven-dried at 60 °C for 72 h before being ground (Retsch MM200) and analysed at the Cornell Isotope Laboratory (COIL, USA) by isotope ratio mass spectrometry (IRMS, Delta V, Thermo Fisher Scientific).

### Microplastic extraction

Each sample consisted of 10 g of angling bait, with 10 replicates analysed for each angling bait product (Supplementary Table S1), resulting in 160 analysed samples. Depending upon the packaging of angling baits and the number of bags purchased (Supplementary Table S1), samples were collected to maximise the number of bags used. When several samples were collected in the same bag, they were collected in different locations within the bag. Samples were first gently ground and homogenized with a mortar and pestle, and then incubated in glass bottles equipped with aluminium caps for 48 h with hydrogen peroxide (H_2_O_2_ (w/w) (30%)) solution (Merck KGaA, Germany) to digest organic matter. Then, samples were filtered through a 500-µm mesh size polyamide fabric (Nitex, SEFAR, Switzerland) and washed with distilled water and ethanol (70% solution in water). The retained content in the Nitex was stored in polystyrene petri dishes. For each sample, two visual inspections of suspected plastic particles (size range: 700 µm (diagonal of 500 µm mesh) to 5 mm) were performed by the same operator under a stereomicroscope (Leica MZ 75 and Nikon SMZ 800). Identified particles were then individually photographed using a binocular magnifier optical (Leica MZ16) equipped with a digital camera (DP20, Olympus, Japan), with their size (i.e. maximal length) measured using ImageJ v.1.8.0^57^. Their colour was defined following previously described literature^58^. Particles were categorised as ‘fibres’ (having at least one very small dimension) or ‘fragments’ (angular and solid or flexible), as adapted from Horton et al.^59^. Each particle was then weighed (AT21 Comparator, d = 0.001 mg, MettlerToledo); there were four particles of mass < 0.001 mg that were not included in calculations of mass concentration. The polymer composition of particles was assessed by attenuated-total-reflectance (ATR) Fourier-transformed infrared spectroscopy (FTIR) equipped with a diamond crystal (Thermo Nicolet 6700, Thermo Fisher Scientific). The crystal was cleaned with ethanol prior to the analysis of each particle and a background was collected at each set of four particles. The IR spectra was obtained with a resolution of 4 cm^−1^ and through the application of 32 scans over the wave-number range of 400 to 4000 cm^−1^. The ATR correction was applied to the spectra, which was then compared to available commercial libraries (OMNIC Software, Thermo Fisher Scientific). A correlation factor of 0.6 was used as threshold to assign composition to the particle. When below this threshold, particles were considered as unidentified and removed from subsequent analyses. Identified particles were classified as microplastic with polymer category (or its artificial additive) following the Polymer Properties Database^60^ when possible, or as non-plastic (Supplementary Figure S1). The following polymer categories were considered: polyethylene, artificial additives (mainly olefin based or alkyd resin, such as lubrifiants and oils^49,61,62^), polyvinylester, polypropylene and polyacrylate. The size range was selected to optimise recovery during the stereomicroscope analyses and an unequivocal identification of particle composition^56,63^.

### Quality control and contamination assessment

All procedures were performed under a fume-hood and metal- and/or glass-ware were used wherever possible. Nitrile gloves and cotton lab coats were always worn. The solvents, distilled water, ethanol and H_2_O_2_ solution were previously filtered through a polyethersufone membrane of 8 µm mesh size (Sterlitech, EUA) to avoid external contamination. The original packaging of each angling bait was sampled and submitted to the same ATR-FTIR analysis to determine its polymer composition. A total of 10 replicates of silica powder (50 µm, Interchim), with around 10 g each, were used as blanks and submitted to the same entire process in the same sample batch. The microscopic inspection indicated the absence of suspicious particles in the size range of this study and contamination was therefore considered negligible.

### Statistical analyses

Microplastic occurrence in angling baits was calculated as the proportion between products containing microplastic and the total products available per category. Microplastic concentration in angling baits was calculated as the number of microplastics per unit of ground bait dry mass (number concentration in MP kg^−1^) and as the mass of microplastics per unit of ground bait dry mass (mass concentration in mg kg^−1^). Fisher exact tests were first used to compare the occurrence of microplastics between angling bait categories and between companies. Generalized linear mixed-effects models (glmm) were used to test the difference in microplastic concentration (number and mass) between the categories of angling baits and between the companies (fixed effects) using angling bait product as a random factor and gamma distribution as family. Fisher exact tests were then used to compare the polymer composition and colour distribution of microplastics between angling bait categories. Spearman correlations tested the relationship between the microplastic concentration (number and mass) in angling baits (averaged value across replicates), number of reported ingredients and C:N ratio (mean value across replicates) across all products (n = 16). All statistical analyses were performed using R v.4.0.2^64^ and generalized linear mixed effects models were performed using the package lme4^64^. Significant levels of generalized mixed effects models were obtained using the ‘Anova’ function in the car package^66^. Error reported around mean values are standard deviation.

## Supplementary Information

Supplementary Information.

## References

[1] Demeneix BA (2020). How fossil fuel-derived pesticides and plastics harm health, biodiversity, and the climate. Lancet Diabetes Endocrinol..

[2] Rochman CM (2018). Microplastics research—from sink to source. Science.

[3] Skalska K, Ockelford A, Ebdon JE, Cundy AB (2020). Riverine microplastics: behaviour, spatio-temporal variability, and recommendations for standardised sampling and monitoring. J. Water Process. Eng..

[4] Dong M (2020). The rapid increases in microplastics in urban lake sediments. Sci. Rep..

[5] Grbić J, Helm P, Athey S, Rochman C (2020). Microplastics entering northwestern Lake Ontario are diverse and linked to urban sources. Water Res..

[6] Dris R (2015). Microplastic contamination in an urban area: a case study in Greater Paris. Environ. Chem..

[7] Mani T, Hauk A, Walter U, Burkhardt-Holm P (2015). Microplastics profile along the Rhine River. Sci. Rep..

[8] Campbell SH, Williamson PR, Hall BD (2017). Microplastics in the gastrointestinal tracts of fish and the water from an urban prairie creek. FACETS.

[9] Pinheiro C, Oliveira U, Vieira M (2017). Occurrence and impacts of microplastics in freshwater fish. J. Aquac. Mar. Biol..

[10] Roch S, Walter T, Ittner LD, Friedrich C, Brinker A (2019). A systematic study of the microplastic burden in freshwater fishes of south-western Germany—are we searching at the right scale?. Sci. Total Environ..

[11] López-Rojo N, Pérez J, Alonso A, Correa-Araneda F, Boyero L (2020). Microplastics have lethal and sublethal effects on stream invertebrates and affect stream ecosystem functioning. Environ. Pollut..

[12] Redondo-Hasselerharm PE, Gort G, Peeters ETHM, Koelmans AA (2020). Nano- and microplastics affect the composition of freshwater benthic communities in the long term. Sci. Adv..

[13] Collard F, Gasperi J, Gabrielsen GW, Tassin B (2019). Plastic Particle Ingestion by Wild Freshwater Fish: A Critical Review. Environmental Science & Technology.

[14] Roch S, Friedrich C, Brinker A (2020). Uptake routes of microplastics in fishes: practical and theoretical approaches to test existing theories. Sci. Rep..

[15] Collard F, Gasperi J, Gabrielsen GW, Tassin B (2019). Plastic particle ingestion by wild freshwater fish: a critical review. Environ. Sci. Technol..

[16] McGoran AR, Cowie PR, Clark PF, McEvoy JP, Morritt D (2018). Ingestion of plastic by fish: a comparison of Thames estuary and firth of Clyde populations. Mar. Pollut. Bull..

[17] Welden NA, Abylkhani B, Howarth LM (2018). The effects of trophic transfer and environmental factors on microplastic uptake by plaice, *Pleuronectes plastessa*, and spider crab *Maja squinado*. Environ. Pollut..

[18] Rochman CM, Hoellein T (2020). The global odyssey of plastic pollution. Science.

[19] Cooke SJ, Cowx IG (2004). The role of recreational fishing in global fish crises. Bioscience.

[20] Arlinghaus R (2020). Global participation in and public attitudes toward recreational fishing: international perspectives and developments. Rev. Fish. Sci. Aquac..

[21] Wolos A, Teodorowicz M, Grabowska K (1992). Effect of ground-baiting on anglers’ catches and nutrient budget of water bodies as exemplified by Polish lakes. Aquac. Fish. Manag..

[22] Arlinghaus, R. Recreational fisheries in Germany—a social and economic analysis, 166. Leibniz-Institute of Freshwater Ecology and Inland Fisheries (IGB). ISSN Nr. 1432–508X (2004).

[23] Arlinghaus R, Mehner T (2003). Socio-economic characterisation of specialised common carp (*Cyprinus carpio* L.) anglers in Germany, and implications for inland fisheries management and eutrophication control. Fish. Res..

[24] Bašić T, Britton R, Jackson MC, Reading P, Grey J (2015). Angling baits and invasive crayfish as important trophic subsidies for a large cyprinid fish. Aquat. Sci..

[25] De Santis V, Roberts CG, Britton JR (2019). Influences of angler subsidies on the trophic ecology of European barbel Barbus barbus. Fish. Res..

[26] Gutmann Roberts C, Bašić T, Amat Trigo F, Britton JR (2017). Trophic consequences for riverine cyprinid fishes of angler subsidies based on marine-derived nutrients. Freshw. Biol..

[27] Amaral SD, Brito D, Ferreira MT, Neves R, Franco A (2013). Modeling water quality in reservoirs used for angling competition: Can groundbait contribute to eutrophication?. Lake Reserv. Manag..

[28] Arlinghaus R, Niesar M (2005). Nutrient digestibility of angling baits for carp, *Cyprinus carpio*, with implications for groundbait formulation and eutrophication control. Fish. Manag. Ecol..

[29] Prata JC (2020). Identification of microplastics in white wines capped with polyethylene stoppers using micro-Raman spectroscopy. Food Chem..

[30] Zhang J, Wang L, Kannan K (2019). Polyethylene terephthalate and polycarbonate microplastics in pet food and feces from the United States. Environ. Sci. Technol..

[31] Akhbarizadeh R (2020). Abundance, composition, and potential intake of microplastics in canned fish. Mar. Pollut. Bull..

[32] Karami A (2018). Microplastic and mesoplastic contamination in canned sardines and sprats. Sci. Total Environ..

[33] Gibb H (2015). Does morphology predict trophic position and habitat use of ant species and assemblages?. Oecologia.

[34] Martin, A. M. Composting of seafood wastes. In *Maximising the Value of Marine By-Products* 486–515 (Elsevier, 2007). 10.1533/9781845692087.3.486.

[35] Dantas DV, Barletta M, da Costa MF (2012). The seasonal and spatial patterns of ingestion of polyfilament nylon fragments by estuarine drums (Sciaenidae). Environ. Sci. Pollut. Res..

[36] Pegado TSS (2018). First evidence of microplastic ingestion by fishes from the Amazon River estuary. Mar. Pollut. Bull..

[37] Ramos J, Barletta M, Costa M (2012). Ingestion of nylon threads by Gerreidae while using a tropical estuary as foraging grounds. Aquat. Biol..

[38] Ryan MG, Watkins L, Walter MT (2019). Hudson River juvenile Blueback herring avoid ingesting microplastics. Mar. Pollut. Bull..

[39] Garcia F (2021). Stable isotope insights into microplastic contamination within freshwater food webs. Environ. Sci. Technol..

[40] Jabeen K (2017). Microplastics and mesoplastics in fish from coastal and fresh waters of China. Environ. Pollut..

[41] Merga LB, Redondo-Hasselerharm PE, Van den Brink PJ, Koelmans AA (2020). Distribution of microplastic and small macroplastic particles across four fish species and sediment in an African lake. Sci. Total Environ..

[42] Park T-J (2020). Occurrence of microplastics in the Han River and riverine fish in South Korea. Sci. Total Environ..

[43] Warrack S, Challis JK, Hanson ML, Rennie MD (2017). Microplastics flowing into lake winnipeg: densities, sources, flux, and fish exposures. Proc. Manit. Undergrad. Sci. Eng. Res..

[44] Banaee M (2019). Evaluation of single and combined effects of cadmium and micro-plastic particles on biochemical and immunological parameters of common carp (*Cyprinus carpio*). Chemosphere.

[45] Nematdoost Haghi B, Banaee M (2017). Effects of micro-plastic particles on paraquat toxicity to common carp (*Cyprinus carpio*): biochemical changes. Int. J. Environ. Sci. Technol..

[46] Xia X (2020). Polyvinyl chloride microplastics induce growth inhibition and oxidative stress in *Cyprinus carpio* var. larvae. Sci. Total Environ..

[47] Hartmann NB (2019). Are we speaking the same language? Recommendations for a definition and categorization framework for plastic debris. Environ. Sci. Technol..

[48] Pazos RS, Maiztegui T, Colautti DC, Paracampo AH, Gómez N (2017). Microplastics in gut contents of coastal freshwater fish from Río de la Plata estuary. Mar. Pollut. Bull..

[49] PlasticsEurope. The facts - 2020. *An analysis of European plastics production, demand and waste data,* France: PlasticsEurope (2020).

[50] Hofland A (2012). Alkyd resins: from down and out to alive and kicking. Prog. Org. Coat..

[51] Karbalaei S (2020). Analysis and inorganic composition of microplastics in commercial Malaysian fish meals. Mar. Pollut. Bull..

[52] Hanachi P, Karbalaei S, Walker TR, Cole M, Hosseini SV (2019). Abundance and properties of microplastics found in commercial fish meal and cultured common carp (*Cyprinus carpio*). Environ. Sci. Pollut. Res..

[53] Jung J-W (2021). Ecological risk assessment of microplastics in coastal, shelf, and deep sea waters with a consideration of environmentally relevant size and shape. Environ. Pollut..

[54] Rødland ES (2020). Road de-icing salt: assessment of a potential new source and pathway of microplastics particles from roads. Sci. Total Environ..

[55] Siegfried M, Koelmans AA, Besseling E, Kroeze C (2017). Export of microplastics from land to sea. A modelling approach. Water Res..

[56] Filella M (2015). Questions of size and numbers in environmental research on microplastics: methodological and conceptual aspects. Environ. Chem..

[57] Rasband WS (1997). ImageJ.

[58] Mani T, Burkhardt-Holm P (2019). Seasonal microplastics variation in nival and pluvial stretches of the Rhine River—from the Swiss catchment towards the North Sea. Sci. Total Environ..

[59] Horton AA, Svendsen C, Williams RJ, Spurgeon DJ, Lahive E (2017). Large microplastic particles in sediments of tributaries of the River Thames, UK—abundance, sources and methods for effective quantification. Mar. Pollut. Bull..

[60] Polymer Properties Database. Polymer Database*.* http://polymerdatabase.com/home.html (2020).

[61] Song YK (2014). Large accumulation of micro-sized synthetic polymer particles in the sea surface microlayer. Environ. Sci. Technol..

[62] Su L (2020). Superimposed microplastic pollution in a coastal metropolis. Water Res..

[63] Christensen ND (2020). Transport and characterization of microplastics in inland waterways. J. Water Process Eng..

[64] R Core Team (2019). R: A Language and Environment for Statistical Computing.

[65] Bates D, Mächler M, Bolker B, Walker S (2015). Fitting Linear mixed-effects models using lme4. J. Stat. Softw..

[66] Fox J, Weisberg S (2019). An R Companion to Applied Regression.

