# Supplementary material for: Microplastic in angling baits as a cryptic source of contamination in European freshwaters
Source: Sci Rep. 2021 May 27;11:11255. doi: 10.1038/s41598-021-90468-0 (PMC8160005; doi:10.1038/s41598-021-90468-0)
Supplement: Supplementary file 1 — Supplementary Information. [file 41598_2021_90468_MOESM1_ESM.pdf]

Supplementary Information

Microplastic in angling baits as a cryptic source of contamination in European freshwaters

Aline Reis de Carvalho[1,2*], Alexis Imbert[1], Ben Parker[3], Axelle Euphrasie[1], Stéphanie Boulêtreau[4], J. Robert Britton[3], Julien Cucherousset[1]

[1] CNRS, Université Toulouse III - Paul Sabatier, IRD, UMR 5174 Laboratoire Évolution et Diversité Biologique (EDB), 118 route de Narbonne, 31062 Toulouse, France

[2] CNRS, Université Toulouse III - Paul Sabatier, UMR 5623 Laboratoire des Interactions Moléculaires et Réactivité Chimique et Photochimique (IMRCP), 118 route de Narbonne, 31062 Toulouse, France

[3] Department of Life and Environmental Sciences, Faculty of Science and Technology, Bournemouth University, Fern Barrow, BH12 5BB Poole, Dorset, UK

[4] CNRS, Université Toulouse III - Paul Sabatier, UMR 5245 Laboratoire Écologie Fonctionnelle et Environnement, 118 route de Narbonne, 31062, Toulouse, France

* Corresponding author: carvalho@chimie.ups-tlse.fr

Supplementary Table S1. List and characteristics of the angling baits analyzed in the present study.

Category	Product	Company	Bags purchased (n)	Bag mass (kg)	Reported ingredients	C:N ratio (mean ± sd) (n =3)	MP concentration (mean ± sd) ($MP.kg^{-1}$ / $mg.kg^{-1}$) (n = 10)
**Groundbaits**	G1	A	1	5	6	23.91 ± 0.92	59.8 ± 84.1 / 7.99 ± 13.9
	G2	B	2	2.5	3	23.70 ± 1.79	60.0 ± 96.6 / 6.92 ± 11.3
	G3	C	5	1	2	24.66 ± 3.58	9.99 ± 31.6 / 2.01 ± 6.35
	G4	A	1	2	10	21.91 ± 0.14	20.0 ± 42.2 / 42.7 ± 90.5
	G5	C	1	1	2	20.52 ± 0.68	0 ± 0 / 0 ± 0
	G6	B	1	0.85	3	22.15 ± 0.33	49.7 ± 52.4 / 25.4 ± 62.0

**Boilies**	B1	A	1	10	0	25.62 ± 1.79	9.93 ± 31.4 / 4.27 ± 13.5
	B2	D	1	5	15	8.82 ± 0.13	49.8 ± 84.6 / 10.7 ± 22.4
	B3	E	1	10	6	20.52 ± 1.42	0 ± 0 / 0 ± 0
	B4	E	1	1	3	7.40 ± 0.06	0 ± 0/ 0 ± 0
	B5	A	1	1	3	23.79 ± 0.19	9.95 ± 31.5 / 5.19 ± 16.4
	B6	F	1	2	8	15.97 ± 0.16	9.67 ± 30.6 / 3.30 ± 10.4
**Pellets**	P1	C	5	0.75	6	8.21 ± 0.42	0 ± 0 / 0 ± 0
	P2	A	1	10	6	7.21 ± 0.08	0± 0 / 0 ± 0
	P3	E	3	2	2	8.22 ± 0.25	0 ± 0 / 0 ± 0
	P4	C	1	1	4	18.12 ± 0.38	0 ± 0 / 0 ± 0

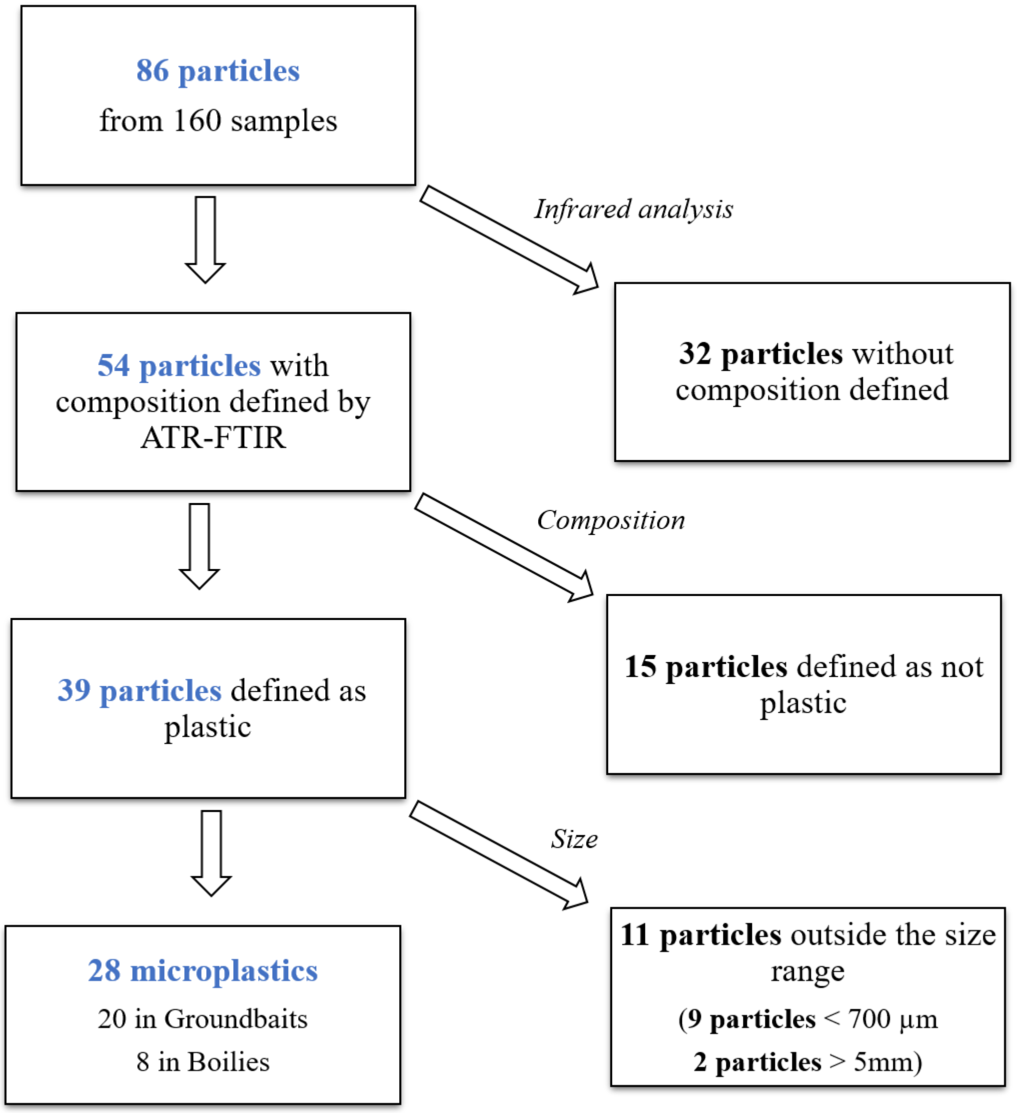

Supplementary Figure S1. Flowchart of microplastic selection for data analyses.

Supplementary Table S2. Polymer composition of angling bait packaging and of microplastics observed in each model.

Category	Product	Packaging composition	Microplastic
**Groundbaits**	G1	polyethylene terephthalate	polypropylene (n = 1), polyvinylester (n = 2), additives (n = 3)
	G2	polyethylene	polyethylene (n = 4), polyvinylester (n = 2)
	G3	polyethylene terephthalate	additive (n = 1)
	G4	polyethylene terephthalate	additives (n = 2)
	G5	polyethylene terephthalate	None
	G6	polyethylene terephthalate	polyethylene (n = 4), additive (n = 1)
**Boilies**	B1	polyethylene	polyethylene (n = 1)
	B2	polyethylene	polyethylene (n = 1), polyvinylester (n = 2) additives (n = 2)
	B3	polyethylene	None
	B4	polyethylene terephthalate	None
	B5	polyethylene terephthalate	polyacrylate (n = 1)
	B6	polyethylene terephthalate	polypropylene (n = 1)
**Pellets**	P1	polyethylene terephthalate	None
	P2	polyethylene	None
	P3	polyethylene terephthalate	None
	P4	polyethylene	None

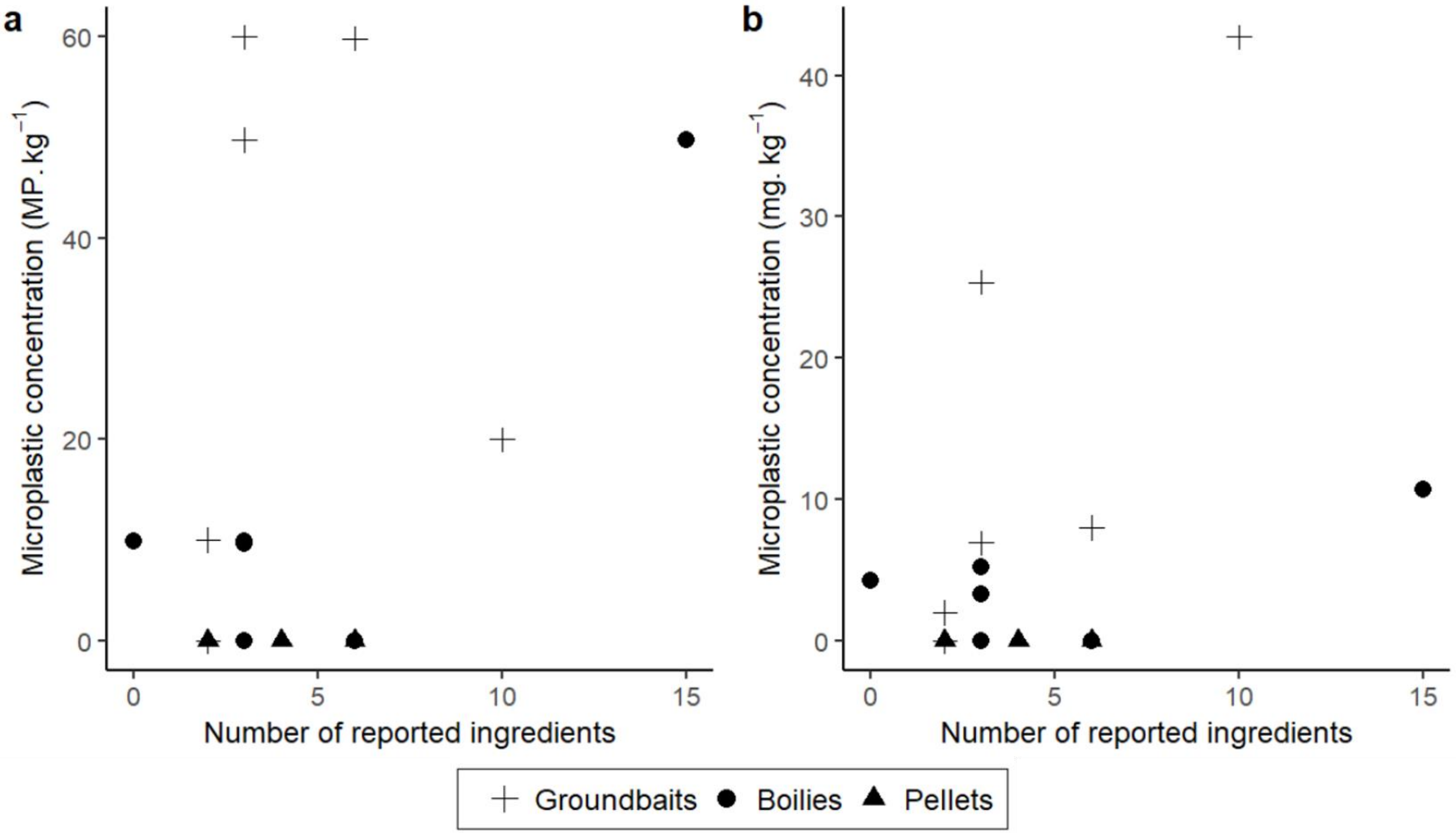

Supplementary Figure S2. Relationship between microplastic concentration in number (a) and mass (b) and number of reported ingredients in angling baits.
